# Real-world effectiveness and protection of SARS-CoV-2 vaccine among patients hospitalized for COVID-19 in Xi’an, China, December 8, 2021, to January 20, 2022: A retrospective study

**DOI:** 10.3389/fimmu.2022.978977

**Published:** 2022-09-23

**Authors:** Xiaowei Li, Yinjuan Xu, Xiaomeng Li, Wenbin Liu, Dan Yao, Weichao Chen, Hongchuan Yu, Langchong He, Shemin Lu, Congshan Jiang, Wenhua Zhu, Liesu Meng

**Affiliations:** ^1^ National Joint Engineering Research Center of Biodiagnostics and Biotherapy, Second Affiliated Hospital, Xi’an Jiaotong University, Xi’an, China; ^2^ Key Laboratory of Environment and Genes Related to Diseases, Ministry of Education, Xi’an Jiaotong University, Xi’an, China; ^3^ Department of Biochemistry and Molecular Biology, Institute of Molecular and Translational Medicine (IMTM), Xi’an Jiaotong University Health Science Center, Xi’an, China; ^4^ Department of Laboratory Medicine, Xi’an Chest Hospital, Xi'an, China; ^5^ Nursing Department, Xi’an Children’s Hospital, Affiliated Children’s Hospital of Xi’an Jiaotong University, Xi’an, China; ^6^ Department of Respiratory Medicine, Xi’an Children’s Hospital, Affiliated Children’s Hospital of Xi’an Jiaotong University, Xi’an, China; ^7^ School of Pharmacy, Xi’an Jiaotong University, Xi’an, China; ^8^ National Regional Children’s Medical Center (Northwest), Key Laboratory of Precision Medicine to Pediatric Diseases of Shaanxi Province, Xi’an Key Laboratory of Children’s Health and Diseases, Shaanxi Institute for Pediatric Diseases, Xi’an Children’s Hospital, Affiliated Children’s Hospital of Xi’an Jiaotong University, Xi’an, China

**Keywords:** SARS-CoV-2, vaccine, COVID-19, clinical features, laboratory findings

## Abstract

**Introduction:**

In December 2021, a large-scale epidemic broke out in Xi’an, China, due to SARS-CoV-2 infection. This study reports the effect of vaccination on COVID-19 and evaluates the impact of different vaccine doses on routine laboratory markers.

**Methods:**

The laboratory data upon admission, of 231 cases with COVID-19 hospitalized from December 8, 2021 to January 20, 2022 in Xi’an, including blood routine, lymphocyte subtypes, coagulative function tests, virus specific antibodies and blood biochemical tests were collected and analyzed.

**Results:**

Of the 231 patients, 21 were not vaccinated, 158 were vaccinated with two doses and 52 with three doses. Unvaccinated patients had a higher proportion of moderate and severe symptoms than vaccinated patients, while two-dose vaccinated patients had a higher proportion than three-dose vaccinated patients. SARS-CoV-2 specific IgG levels were significantly elevated in vaccinated patients compared with unvaccinated patients. Particularly, unvaccinated patients had lower counts and percentages of lymphocytes, eosinophils and CD8^+^ T-lymphocytes, and elevated coagulation-related markers. In addition, vaccination had no effect on liver and kidney function.

**Conclusions:**

Vaccination against SARS-CoV-2, inducing high IgG level and increased CD8^+^ T cells and eosinophils, and regulating coagulation function, can significantly attenuate symptoms of COVID-19, suggesting that the vaccine remains protective against SARS-CoV-2.

## Introduction

Since the first report of severe acute respiratory syndrome coronavirus 2 (SARS-CoV-2) infection in Wuhan in December 2019, the widespread pandemic of Corona Virus Disease 2019 (COVID-19) has become a major health problem all over the world (1). As of May 2022, the cumulative number of global cases has exceeded 526 million, with more than 6.28 million deaths. Under the strict massive prevention and quarantine policies, the number of COVID-19 cases in China has decreased rapidly. However, there are still localized SARS-CoV-2 infections and epidemics in China. On December 8, 2021, the first local confirmed case of COVID-19 was reported in Xi’an, China. On December 26, 2021, the Shaanxi Province New Coronary Pneumonia Epidemic Prevention and Control Press Conference reported officially that the Provincial Center for Disease Control and Prevention conducted whole-genome sequencing of 36 samples from COVID-19 patients, and all were Delta variants (http://www.shaanxi.gov.cn/szf/xwfbh/202112/t20211226_2205519.html). Through massive prevention and control management, there were “zero new cases” of confirmed cases of COVID-19 in Xi’an until January 20, 2022. Although not all samples have been verified by sequencing, the epidemiological investigations showed that they were community-transmitted infections, in the relevant transmission chain, suggesting that this outbreak was most likely the transmission of Delta strain. This Xi’an epidemic with a total of 2,050 confirmed cases of COVID-19 is the most severe and extensive in China’s megacities since the outbreak in Wuhan, China.

When cases are on the rise and transmission accelerates, the likelihood of virus undergoing mutation increases (2). Slight changes have little or no impact on their biological properties, such as reproductivity and pathogenicity. However, a few accidental yet possible mutations can still incur more dangerous variants, potentiating epidemics on a much larger scale (3). The B.1.617.2 (Delta) variant of SARA-CoV-2 was first detected in India and expanded rapidly, later becoming the dominant strain in almost every country by the end of 2021 (4). The Delta variant has nine amino acid site mutations in the spike protein, making it more contagious and confers higher pathogenicity and immune escape capability (5). The reproductive number of the Delta variant of SARS-CoV-2 is far higher than the ancestral SARS-CoV-2 virus (6). Luo, C. et al. speculates that increased fitness of the Delta variant causes more frequent recovery of infectious virus in fully vaccinated individuals, which may be associated with SARS-CoV-2 infections and lead to higher viral loads and worse disease outcomes (7).

Vaccination is an effective tool in a widespread pandemic and protects against severe disease and high mortality (8). Up to now, more than one hundred COVID-19 vaccines have been developed (9), such as Pfizer mRNA vaccine (10)and Sinovac inactivated vaccine (11). Each vaccine approved by WHO has undergone a range of rigorous clinical trials to test its safety and quality (12). COVID-19 mRNA vaccines deliver the viral RNA to the target cells in the host through a vector, and the target protein or immunogen, when expressed, can activate the immune response against viral infection (10). Inactivated vaccines are prepared by using completely inactivated SARS-CoV-2 or recombinant spike protein, which can induce virus-neutralizing antibodies to potentiate protection against virus (11). It is worth noting that vaccination with enough doses may induce strong protection in most people, but exceptions do exist (13). This explains the infection among fully vaccinated populations, and therefore defining vaccine efficacy is urgent and critical (14). Moreover, some variants may also affect the vaccination and the consequent immunity against other virus infections, as exemplified by the emergence of the SARS-CoV-2 Delta variant narrows the differences in COVID-19 infection rates between vaccinated and unvaccinated populations (15). However, vaccines remain protective against the variants because of the broad immune response they cause, especially in preventing severe symptoms, hospitalization, and death (16). During the epidemic in Xi’an, most citizens had already received 2 or 3 doses of the SARS-CoV-2 vaccine. However, there are still a few unvaccinated citizens. Therefore, the epidemic in Xi’an offers an opportunity to further understand the vaccine efficacy on SARS-CoV-2 infection and COVID-19 protection.

In this context, the retrospective, single-center study aimed to analyze routine laboratory markers in confirmed cases of COVID-19 and compare the difference between unvaccinated and vaccinated cases.

## Methods

### Research design and participant inclusion

We designed a retrospective study that included the collection and analysis of data from 261 patients with COVID-19 who were hospitalized in Xi’an Chest Hospital from December 8, 2021 to January 20, 2022. Of these patients, 13 patients younger than 18 years and 17 patients for whom some laboratory data were missing were excluded. The remaining 231 patients with all laboratory tests are included in this study. Laboratory confirmation of SARS-CoV-2 infection was established on the positive results of real-time reverse transcription-polymerase chain reaction (RT-PCR) test of nasopharyngeal swabs. All research subjects were diagnosed following the Diagnosis and Treatment Protocol for Novel Coronavirus Pneumonia, and classified as mild cases (mild clinical symptoms with no sign of pneumonia on imaging), moderate cases (showing fever and respiratory symptoms with imaging evidence of pneumonia) and severe cases (adult cases meeting any of the following criteria: (1) Respiratory distress (≥30 breaths/min); (2) Oxygen saturation ≤ 93% at rest; (3) Arterial partial pressure of oxygen (PaO_2_)/fraction of inspired oxygen (FiO_2_) ≤ 300 mmHg (1 mmHg = 0.133 kPa) (17). All patients were grouped according to the dose of vaccination for subsequent statistical analysis and comparison.

### Data collection

Data including demographic information, clinical features and laboratory indexes were collected from patients’ medical records. Demographic and clinical features include gender, age, chronic disease history and so on. We collected the laboratory data upon admission, including blood routine, lymphocyte subtypes, coagulative function tests, antigen-specific IgG, IgM, blood biochemical tests and so on. Blood routine examination can usually be divided into three categories: red blood cell (RBC), leukocyte, and platelet. The RBC-related parameters include RBC counts, hemoglobin (Hb), hematocrit (HCT), mean corpuscular volume (MCV), mean corpuscular hemoglobin (MCH), mean corpuscular hemoglobin concentration (MCHC), red blood cell distribution width (RDW) and erythrocyte sedimentation rate (ESR). Leukocyte system analysis includes absolute numbers and percentages of neutrophils, eosinophils, basophils, lymphocytes, and monocytes. The platelet-related parameters include platelet counts, mean platelet volume (MPV), and platelet distribution width (PDW).

Lymphocyte subtype analysis includes absolute numbers and percentages of B-lymphocytes, natural killer (NK) cells, T-lymphocytes, and their subtypes. Coagulative function indexes include prothrombin time (PT), activated partial thromboplastin time (APTT), thrombin time (TT), fibrinogen (FBG), international normalized ratio (INR), D-dimer (D-D), and fibrinogen degradation products (FDP).

In the blood biochemical test indicators, we collected liver function and renal function tests. Liver function tests include γ-glutamyl transferase (GGT), alanine aminotransferase (ALT), aspartate aminotransferase (AST), alkaline phosphatase (ALP), bilirubin, total protein (TP), albumin/globulin (A/G), adenosine deaminase (ADA), total biliary acid (TBA), lactate dehydrogenase (LDH) and superoxide dismutase (SOD). Renal function tests include serum potassium (K), serum sodium (Na), serum chloride (CL), serum calcium (Ca), Urea nitrogen (UN), uric acid (UA), and creatinine (Cr).

### Sample collection and laboratory testing procedures

Blood samples were drawn from peripheral veins for each patient infected with SARS-CoV-2 virus. The blood routine test is completed by the automatic blood cell analyzer (BC-6800Plus, Mindray, China). Lymphocyte subtypes were analyzed by a flow cytometer (Canto II, BD Biosciences, USA) using 4-colour BD Multitest™ IMK kit, including T lymphocytes (CD3^+^), B lymphocytes (CD19^+^), helper/inducer T lymphocytes (CD3^+^CD4^+^), suppressor/cytotoxic T lymphocytes (CD3^+^CD8^+^), and natural killer (NK) lymphocytes (CD3^–^CD16^+^ and/or CD56^+^). D-dimer and FDP were analyzed with the blood coagulation analyzer (ACL TOP 700, Werfen, Spain).

SARS-CoV-2 IgG was detected by indirect ELISA using the magnetic particle chemiluminescence immunoassay method (Maccura Biotechnology). Samples were mixed with magnetic particles coated with the recombinant SARS-CoV-2 antigen, in which the specific IgG or antibody could combine with the recombinant antigen to form an immune complex, and then incubated with acridine ester-labeled mouse anti-human IgG antibody. Substrate solution was added and the chemiluminescence results were detected by an automatic chemiluminescence immunoassay i1000 analyzer (Maccura Biotechnology, China).

SARS-CoV-2 IgM was also detected by using an ELISA kit purchased from Maccura Biotechnology. Samples were mixed with magnetic particles coated with mouse anti-human IgM monoclonal antibody, and the IgM antibody in the sample were captured to form an immune complex. Then acridine ester-labeled SARS-CoV-2 antigen was added followed by the substrate solution. Chemiluminescence results were detected by an automatic chemiluminescence immunoassay i1000 analyzer (Maccura Biotechnology, China).

### Statistical analysis

Statistical analysis and graph construction were conducted using GraphPad Prism software 8 (GraphPad, La Jolla, California, USA). The continuous variables were expressed by median (interquartile, IQR). The differences among groups were analyzed using the One-way ANOVA and *post hoc* Bonferroni test for continuous variables normally distributed and using Kruskal-Wallis and *post hoc* Dunn’s test for the quantitative data of non-normal distribution. The differences in proportions for categorical variables were analyzed using the *chi*-square test or fisher’s exact test. *P* value of less than 0.05 is considered significant. *P* values between 0.01 and 0.05, 0.001 and 0.01, 0.0001 and 0.001, and < 0.0001 were considered statistically significant (*), very significant (**), extremely significant (***) and super significant (****).

## Results

### Demographic and clinical characteristics of COVID-19 patients

A total of 231 inpatients admitted to Xi’an Chest Hospital with a diagnosis of COVID-19 were included in the retrospective study. The median age was 37 years (IQR, 28-48; range, 18-85 years) and 123 (53.2%) were male. Of the 231 patients, 21 (9.1%) were unvaccinated, 158 (68.4%) had received two doses, and 52 (22.5%) had received three doses. The vaccines immunized by these patients included two types, inactivated vaccines produced by China National Biotec Group Company Limited (CNBG) and Sinovac Biotech Ltd, and recombinant antigen protein of SARS-CoV-2 vaccines (CHO cells) produced by Anhui Zhifei Longcom Biopharmaceutical. Among the patients who received the vaccine, 48% used the inactivated vaccines produced by Sinovac Biotech only, 29.6% by CNBG only, 12.8% by both Sinovac Biotech and CNBG, and the rest 9.6% patients used the recombinant protein vaccines produced by Anhui Zhifei Longcom Biopharmaceutical.

Accordingly, these patients were divided into three groups, unvaccinated, two-dose vaccinated, and three-dose vaccinated. No significant difference was observed in the patient ages of the three groups. Among all patients, 38 (16.5%) patients suffered from underlying diseases, including hypertension (10.4%), cardiovascular disease (3.9%), diabetes (3.0%), chronic liver disease (2.6%), cerebrovascular disease (0.9%) and chronic obstructive pulmonary disease (0.4%). Compared with the two-dose vaccinated and three-dose vaccinated groups, the proportion of patients with a history of diabetes was higher in the unvaccinated group (*P*1 = 0.0018, *P*2 = 0.0221). All demographic characteristics and clinical manifestations of the patients are shown in **Table 1**.

**Table 1:** Demographic and baseline characteristics of patients with COVID-19.

	Total (n = 231)	Unvaccinated (n = 21)	Two doses vaccinated (n = 158)	Three doses vaccinated (n = 52)	*P*-value
**Characteristics**					
Age, median (IQR), range	37 (28-48), 18-85	38 (32-54.5), 25-85	36 (26.75-48.25), 18-78	37 (30.25-45.75), 19-67	*P*1 = 0.1449, *P*2 = 0.2870, *P*3 = 0.7804
**Sex**					
Male (%)	123 (53.2)	10 (47.6)	81 (51.3)	32 (61.5)	
Female (%)	108 (46.8)	11 (52.4)	77 (48.7)	20 (38.5)	
**Chronic medical illness**					
Any (%)	38 (16.5)	6 (28.6)	26 (16.5)	6 (11.5)	*P*1 = 0.2213, *P*2 = 0.0911, *P*3 = 0.5065
Chronic obstructive pulmonary disease (%)	1 (0.4)	0 (0)	1 (0.6)	0 (0)	*P*1>0.9999, *P*2>0.9999, *P*3>0.9999
Hypertension (%)	24 (10.4)	5 (23.8)	14 (8.9)	5 (9.6)	*P*1 = 0.0526, *P*2 = 0.1388, *P*3>0.9999
Cardiovascular disease (%)	9 (3.9)	3 (14.3)	5 (3.2)	1 (1.9)	*P*1 = 0.0532, *P*2 = 0.0690, *P*3>0.9999
Cerebrovascular disease (%)	2(0.9)	1 (4.8)	1 (0.6)	0 (0)	*P*1 = 0.2215, *P*2 = 0.2877, *P*3>0.9999
Chronic liver disease (%)	6 (2.6)	0 (0)	6 (3.8)	0 (0)	*P*1>0.9999, *P*2>0.9999, *P*3 = 0.3399
Diabetes (%)	7 (3.0)	4 (19)	2 (1.3)	1 (1.9)	**P1 = 0.0018, *P2 = 0.0221, *P*3>0.9999
**Signs and symptoms**					
Fever (%)	95 (41.1)	9 (42.8)	65 (41.1)	21 (40.4)	*P*1>0.9999, *P*2>0.9999, *P*3 = 0.3399
Dry cough (%)	67 (29.0)	5 (23.8)	50 (31.6)	12 (23.1)	*P*1 = 0.6165, *P*2>0.9999, *P*3 = 0.2941
Expectoration (%)	59 (25.5)	7 (33.3)	43 (27.2)	9 (17.3)	*P*1 = 0.6068, *P*2 = 0.2095, *P*3 = 0.1950
Rhinorrhea (%)	65 (28.1)	4(19.0)	52 (32.9)	9 (17.3)	*P*1 = 0.3156, *P*2>0.9999, *P3 = 0.0350
Hyposmia (%)	27 (11.7)	2 (9.5)	18 (11.4)	7 (13.5)	*P*1 = 0.6068, *P*1 = 0.2095, *P*9 = 0.1950
Loss of taste (%)	14 (6.1)	1 (4.8)	9 (5.7)	4 (7.7)	*P*1>0.9999, *P*2>0.9999, *P*3 = 0.7400
Shortness of breath (%)	20 (8.7)	3 (14.3)	13 (8.2)	4 (7.7)	*P*1 = 0.4076, *P*2 = 0.4026, *P*3>0.9999
Myalgia (%)	17 (7.4)	1 (4.8)	15 (9.5)	1 (1.9)	*P*1 = 0.6978, *P*2 = 0.4954, *P*3 = 0.1270
Headache (%)	15 (6.5)	2 (9.5)	13 (8.2)	0 (0)	*P*1 = 0.6902, *P*2 = 0.0799, *P3 = 0.0413
Dizziness (%)	4 (1.7)	1 (4.8)	3 (1.9)	0 (0)	*P*1 = 0.3957, *P*2 = 0.2877, *P*3>0.9999
Fatigue (%)	27 (11.7)	2 (9.5)	18 (11.4)	7 (13.5)	*P*1>0.9999, *P*2>0.9999, *P*3 = 0.8051
Pharyngalgia (%)	45 (19.5)	5 (23.8)	26 (16.5)	14 (26.9)	*P*1 = 0.3714, *P*2>0.9999, *P*3 = 0.1059
Pharyngeal stem (%)	57 (24.7)	3 (14.3)	43 (27.2)	11 (21.1)	*P*1 = 0.2893, *P*2 = 0.7439, *P*3 = 0.4660
Anorexia (%)	15 (6.5)	4 (19.0)	9 (5.7)	2 (3.8)	*P1 = 0.0498, *P*2 = 0.0532, *P*3>0.9999

Categorical variables were summarized as counts (percentages), and analyzed by fisher’s exact test.

P1: Unvaccinated vs. Two doses vaccinated, P2: Unvaccinated vs. Three doses vaccinated, P3: Two doses vaccinated vs. Three doses vaccinated.

*P < 0.05, ** P < 0.01.

**Figure 1:**
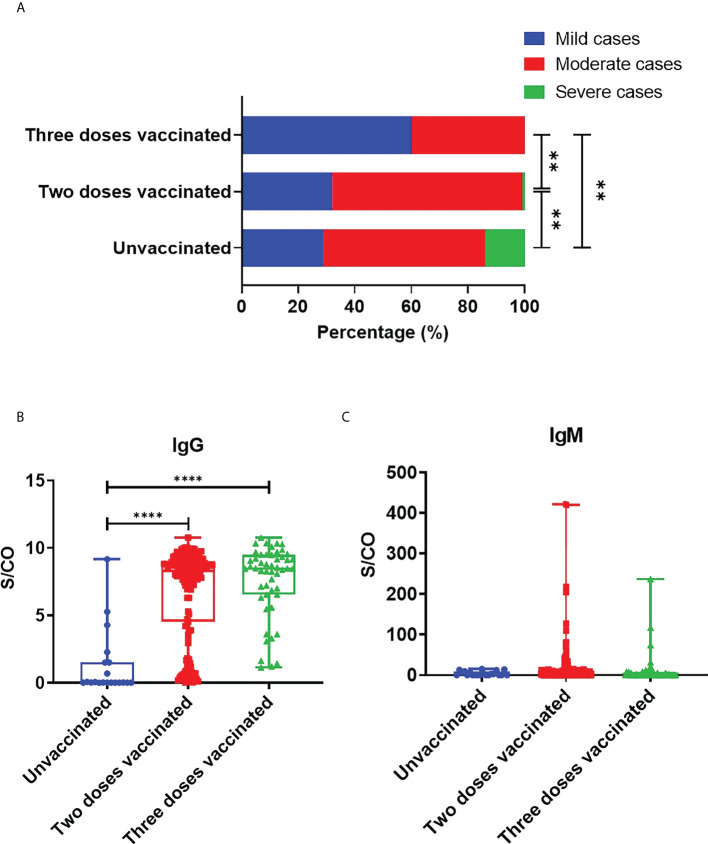
Protection of SARS-CoV-2 vaccine against COVID-19. **(A)** COVID-19 disease severity in adult patients with different doses of vaccine. Statistical significance was determined by Chi-square test. B,C. S/CO values of IgG **(B)** and IgM **(C)** in unvaccinated, two-dose vaccinated and three-dose vaccinated patients. Statistical significance was determined by Kruskal-Wallis test and *post hoc* Dunn’s test. **P < 0.01, ****P < 0.0001.

In total, 87 (37.6%) out of 231 patients were categorized into mild type cases, 139 patients (60.2%) into moderate type cases, and 5 (2.2%) into severe type cases. Among the 5 severe type patients, 3 were not vaccinated, and 2 received two doses of vaccines. The 5 severe type patients were all above the age of 60 and had underlying diseases, such as hypertension and cardiovascular disease. Compared with the unvaccinated group (28.6%), the proportion of mild type patients in two-dose vaccinated group (31.6%), and three-dose vaccinated group (59.6%) was significantly higher (**Figure 1A**). At the same time, compared with the two-dose vaccinated group, the three-dose vaccinated group had a higher proportion of mildly symptomatic cases, with a significant difference (*P*=0.0014). As for the symptoms and signs of the patients, the most common symptoms are fever (41.1%), dry cough (29.0%), rhinorrhea (28.1%), expectoration (25.5%), pharyngeal stem (24.7%), pharyngalgia (19.5%), fatigue (11.7%) and hyposmia (11.7%). More detailed clinical information is listed in **Table 1**. In addition, we analyzed the time from symptom onset to hospital admission and found no significant difference among the three groups (**Figure S1A**).

### Vaccine immunogenicity

We collected the results of SARS-CoV-2 specific antibodies from all COVID-19 patients, including IgG and IgM types. We analyzed the time from admission to SARS-CoV-2 antibody detection in patients, and the results showed no significant difference among the three groups (**Figure S1B**). The mean S/CO values of IgG in patients’ sera vaccinated with zero, two, and three doses were respectively 1.247, 6.753, and 7.622. Compared with unvaccinated patients, the levels of IgG against SARS-CoV-2 in two-dose vaccinated (*P*<0.0001) and three-dose vaccinated group (*P*<0.0001) were significantly elevated, while there were no significant differences between the two-dose and three-dose vaccinated groups (*P*=0.0929) (**Figure 1B**). The above data demonstrated that vaccination could effectively induce humoral immune responses and specific IgG production. IgM usually plays a role in early infection. Our results showed that the mean S/CO values of serum IgM in unvaccinated, two-dose vaccinated, and three-dose vaccinated groups were respectively 4.19, 15.32, and 11.14. IgM levels seemed to have no marked difference between unvaccinated and vaccinated patients. But it is worth noting that the IgM levels of the patient who received three doses were lower than those who received two doses (**Figure 1C**).

### The laboratory indexes of red blood cells, platelets, and coagulation function of COVID-19 patients

We analyzed the laboratory indexes of red blood cells (RBC counts, hemoglobin, HCT, MCV, MCH, MCHC, RDW, ESR), platelets (platelets counts, MPV, PDW), and coagulation function (APTT, PT, TT, FBG, INR, D-dimer, FDP) (**Table 2**). There was no significant difference in the level of RBC, platelets and their related indexes. Most coagulation-related markers appeared to be elevated to varying degrees in unvaccinated patients. APTT (*P*=0.0002) and TT (*P*=0.0071) values were significantly higher in unvaccinated patients compared with twice-vaccinated patients. The values of APTT (*P*=0.0007) and FBG (*P*=0.0176) were significantly elevated in unvaccinated patients compared with triple vaccinated patients. Moreover, we found the values of FBG (*P*=0.0091) were significantly lower in the three doses vaccinated groups compared to the two doses vaccinated group. The above results suggest that the vaccine significantly impacts coagulative function in COVID-19 patients.

**Table 2:** Red blood cells, platelets and coagulation function analysis in patients with COVID-19.

	NormalRange	Total (n = 231)	Unvaccinated (n = 21)	Two doses vaccinated (n = 158)	Three doses vaccinated (n = 52)	*P*-value	
**Red blood cells and platelets**							
Red blood cell counts (RBC), ×10^12^/L	Male: 4.3-5.8 Female: 3.8-5.1	4.48 (4.14-4.89)	4.51 (4.115-5.435)	4.475 (4.148-4.853)	4.45 (4.13-4.928)	*P*=0.4548	
Hemoglobin (Hb), g/L	Male: 130-175 Female: 115-150	139 (123-153)	131 (118-160.5)	139 (123.8-152)	142 (120.5-152.3)	*P*=0.9014	
Hematocrit (HCT), %	Male: 40.0-50.0 Female: 35.0-45.0	41.5 (37.1-45.1)	38.1 (36-48.35)	41.3 (37.18-44.9)	41.9 (37.53-45.6)	*P*=0.9446	
Mean corpuscular volume (MCV), fl	82.0-100.0	91.9 (88.7-94.3)	89.5 (85.8-92.7)	92.45 (89.18-94.5)	91.9 (88.43-95.75)	*P=0.0470	*P*1 = 0.0566, *P*2 = 0.0589, *P*3>0.9999
Mean corpuscular hemoglobin (MCH), Pg	27.0-34.0	30.8 (29.8-31.9)	30 (28.95-30.7)	30.9 (29.9-31.9)	31 (29.7-32.18)	*P*=0.0785	
Mean corpuscular hemoglobin concentration (MCHC), g/L	316-354	334 (328-339)	333 (327-336)	334 (329-339.3)	337 (327.3-339)	*P*=0.4435	
Red blood cell distribution width (RDW), fl	35.0-56.0	41 (39.8-42.9)	42.4 (40.55-43.2)	40.85 (39.68-42.8)	41.15 (39.65-42.85)	*P*=0.2255	
Platelet counts, ×10^9^/L	125-350	200 (159-237)	191 (162-221.5)	200 (150.8-243.5)	209 (171.3-241.3)	*P*=0.5573	
Mean platelet volume (MPV), fl	6.8-13.5	10.6 (9.7-11.5)	10.1 (9.1-11.3)	10.4 (9.7-11.43)	11 (9.725-11.68)	*P*=0.4042	
Platelet distribution width (PDW), fl	10.0-18.0	16.2 (16-16.4)	16.2 (15.7-16.35)	16.2 (16-16.4)	16.3 (16-16.5)	*P=0.0351	*P*1 = 0.4188, *P*2 = 0.413, *P*3 = 0.1922
Erythrocyte sedimentation rate (ESR), mm/h	Male:0-15 Female:0-20	24 (13-40)	22 (12-39.75)	25 (14-40)	23 (9-35)	*P*=0.3302	
**Coagulation function**							
Prothrombin time (PT), s	11.8-15.1	13.5 (12.3-14.4)	13.7 (12.5-14.85)	13.4 (12.2-14.33)	13.6 (12.4-14.4)	*P*=0.7692	
Activated partial thromboplastin time (APTT), s	25.4-38.4	31.9 (29.5-33.7)	33.3 (32-37.65)	31.5 (29.5-33.53)	31.2 (28.9-33.68)	***P=0.0002	***P1 = 0.0002, ***P2 = 0.0007, *P*3>0.9999
Thrombin time (TT), s	11.0-17.8	14.9 (14.1-15.7)	15.9 (14.85-16.8)	14.7 (13.9-15.43)	15.05 (14.4-15.9)	**P=0.0055	**P1 = 0.0071, *P*2 = 0.2361, *P*3 = 0.3283
Fibrinogen (FBG), g/L	2.20-4.96	3.31 (2.82-3.87)	3.73 (2.925-4.07)	3.35 (2.865-3.97)	3 (2.665-3.5)	**P=0.0038	*P*1 = 0.9061, *P2 = 0.0176, **P3 = 0.0091
International normalized ratio (INR)	0.70-1.40	1.06 (0.94-1.14)	1.07 (0.96-1.185)	1.05 (0.94-1.133)	1.06 (0.95-1.14)	*P*=0.7671	
D-dimer (D-D), µg/mL	0-1.00	0.42 (0.36-0.54)	0.49 (0.36-0.59)	0.435 (0.37-0.55)	0.39 (0.3125-0.4675)	*P*=0.3650	
Fibrinogen degradation products (FDP), µg/mL	0-5.00	1.9 (1.5-2.4)	1.9 (1.7-2.8)	1.9 (1.5-2.4)	1.7 (1.5-1.9)	*P*=0.3254	

Continuous variables were shown in median (interquartile ranges). The Kruskal-Wallis test and post hoc Dunn’s test were used to analyze for the quantitative data of non-normal distribution among three groups. The One-way ANOVA and post hoc Bonferroni test were used to analyze for continuous variables normally distributed among three groups.

P: for three groups, P1: Unvaccinated vs. Two doses vaccinated, P2: Unvaccinated vs. Three doses vaccinated, P3: Two doses vaccinated vs. Three doses vaccinated.

*P < 0.05, **P < 0.01, ***P < 0.001.

### Leukocyte subtypes analysis of COVID-19 patients

Various types of leukocytes were analyzed (**Table 3**). Among these cells, eosinophils and lymphocytes differed most significantly among the groups. The absolute number and percentage of eosinophils were significantly different among the three vaccination groups, exhibiting a more stressed decrease in unvaccinated patients. The number and percentage of lymphocytes were also significantly decreased in patients without vaccination compared with those twice or triple vaccinated. The percentage of monocytes was increased in unvaccinated patients compared with twice (*P*=0.0400), or triple vaccinated patients (*P*=0.0404). No significant difference was found in neutrophils and basophils.

**Table 3:** Leukocyte subtypes analysis in patients with COVID-19.

	Normal range	Total (n = 231)	Unvaccinated (n = 21)	Two doses vaccinated (n = 158)	Three doses vaccinated (n = 52)	*P*-value	
**Leukocyte subtypes**							
White blood cells, ×10^9^/L	3.50-9.50	4.825 (3.953-5.858)	4.765 (4.003-5.21)	4.79 (3.97-5.88)	4.89 (3.62-5.925)	*P*=0.4833	
Neutrophils, ×10^9^/L	1.80-6.30	2.86 (2.2-3.775)	3.035 (2.193-3.7)	2.84 (2.283-3.78)	2.97 (2.04-3.81)	*P*=0.8767	
Neutrophil percentage, %	40.0-75.0	60.86 (53.67-69.12)	62.44 (55.67-72.67)	60.86 (54.19-69.07)	59.76 (51.91-67.57)	*P*=0.3684	
Eosinophils, ×10^9^/L	0.02-0.52	0.04 (0.01-0.1)	0.01 (0-0.02)	0.04 (0.01-0.09)	0.06 (0.03-0.1275)	****P <0.0001	*P1 = 0.0045, ***P2 = 0.0001, *P*3 = 0.1130
Eosinophil percentage, %	0.4-8.0	0.9 (0.2-2.1)	0.2 (0.1-0.6)	0.9 (0.275-1.825)	1.1 (0.625-2.8)	**P=0.0098	*P1 = 0.0339, ***P2 = 0.0010, *P*3 = 0.0962
Monocytes, ×10^9^/L	0.10-0.60	0.38 (0.29-0.49)	0.43 (0.37-0.58)	0.37 (0.29-0.5075)	0.38 (0.28-0.47)	*P*=0.3004	
Monocyte percentage, %	3.0-10.0	7.695 (6.338-10.02)	9.515 (7.865-11.66)	7.59 (6.23-9.85)	7.39 (6.27-9.845)	*P=0.0329	*P1 = 0.0400, *P2 = 0.0404, *P*3>0.9999
Basophils, ×10^9^/L	0-0.06	0.01 (0.01-0.02)	0.01 (0.01-0.01)	0.01 (0.01-0.02)	0.01 (0.01-0.02)	*P*=0.3095	
Basophil Percentage, %	0-1.0	0.2 (0.2-0.3)	0.2 (0.15-0.3)	0.2 (0.2-0.3)	0.3 (0.1-0.4)	*P*=0.1646	
Lymphocytes, ×10^9^/L	1.10-3.20	1.31 (0.945-1.755)	0.95 (0.81-1.438)	1.405 (0.94-1.808)	1.34 (1.125-1.71)	*P=0.0372	*P1 = 0.0424, *P2 = 0.0490, *P*3>0.9999
Lymphocyte Percentage, %	20.0-50.0	29.57 (21.59-36.75)	25.21 (16.71-32.84)	29.45 (21.59-36.76)	30.68 (24.75-37.69)	*P*=0.2056	
Neutrophils/lymphocytes ratio (NLR)		2.074 (1.454-3.138)	2.478 (1.754-4.392)	2.080 (1.485-3.134)	2.000 (1.397-2.700)	*P*=0.6505	
**Lymphocyte subtypes**							
B cells,/µL	90-580	134.5 (86.25-195.5)	82 (69-133.5)	137 (90.75-208.5)	136 (104.5-195)	*P=0.0417	*P1 = 0.0397, *P*2 = 0.0816, *P*3>0.9999
B cell percentage, %	5.0-20.0	10.9 (7.45-14.2)	7.9 (6.55-12.35)	11.45 (7.925-14.23)	10.9 (7.95-13.7)	*P*=0.2131	
Natural killer (NK) cells,/µL	150-900	177.5 (133-312.3)	257 (151-365)	155 (125-235)	240 (151.5-339)	*P=0.0253	*P*1 = 0.1790, *P*2>0.9999, *P*3 = 0.0693
NK cell percentage, %	5.0-28.0	15.65 (10.35-22.78)	18.6 (15.3-37.2)	13.2 (9.7-20.15)	17.4 (12.05-26.25)	**P=0.0075	*P1 = 0.0187, *P*2 = 0.7546, *P*3 = 0.1343
T cells,/µL	690-2540	936 (643-1330)	604 (544-942.5)	998 (685.5-1350)	844 (758.5-1414)	*P=0.0339	*P1 = 0.0283, *P*2 = 0.0909, *P*3>0.9999
T cell percentage, %	60.0-84.00	71.05 (66.17-77.96)	63.77 (51.26-69.38)	72.86 (67.24-79.06)	70.15 (62.16-73.81)	***P=0.0010	**P1 = 0.0024, *P2 = 0.2771, *P*3 = 0.0886
**T-cell subtypes**							
CD4^+^ T cells,/µL	410-1590	482.5 (330-667)	330 (316-521.5)	513 (318.3-690.8)	474 (364-687)	*P*=0.1583	
CD4^+^ T cells percentage, %		37.47 (33.21-42.24)	35.42 (32.06-40.37)	37.68 (34.07-42.69)	37.92 (31.12-41.36)	*P*=0.4211	
CD8^+^ T cells,/µL	190-1140	415.5 (267.3-635.3)	227 (205.5-384.5)	429 (301.3-669.3)	421 (320-597.5)	**P=0.0099	**P1 = 0.0077, *P2 = 0.0261, *P*3>0.9999
CD8^+^ T cell percentage, %		31.4 (25.66-37.94)	25.77 (21.28-28.61)	32.37 (26.85-39.11)	32.88 (25.94-37.17)	*P=0.0144	*P1 = 0.0109, *P2 = 0.0484, *P*3>0.9999
CD4/CD8	1.40-2.00	1.18 (0.9-1.543)	1.45 (1.11-1.55)	1.18 (0.9-1.533)	1.14 (0.86-1.6)	*P*=0.3171	
CD4^+^ CD8^+^ T cells,/µL	0-125	9 (6-14)	8 (3-10.5)	9 (5.25-15)	10 (6-14.5)	*P*=0.3686	
CD4^+^ CD8^+^ T cell percentage, %	0-5.00	0.725 (0.44-1.073)	0.69 (0.335-0.81)	0.7 (0.44-1.08)	0.95 (0.53-1.095)	*P*=0.3395	

Continuous variables were shown in median (interquartile ranges). The Kruskal-Wallis test and post hoc Dunn’s test were used to analyze for the quantitative data of non-normal distribution among three groups. The One-way ANOVA and post hoc Bonferroni test were used to analyze for continuous variables normally distributed among three groups.

P: for three groups, P1: Unvaccinated vs. Two doses vaccinated, P2: Unvaccinated vs. Three doses vaccinated, P3: Two doses vaccinated vs. Three doses vaccinated.

*P < 0.05, **P < 0.01, ***P < 0.001, ****P < 0.0001.

We further analyzed different subtypes of lymphocytes. The time from admission to lymphatic subtype analysis of patients was analyzed and found no significant difference among groups (**Figure S1C**). T-lymphocytes were significantly decreased in unvaccinated patients, both in percentage and absolute count. Compared with the twice vaccinated patients, B lymphocyte counts in unvaccinated patients were significantly reduced (*P*=0.0397). The percentage of NK lymphocytes in the twice vaccinated patients was significantly reduced compared with unvaccinated (*P*=0.0187) patients.

As for T-lymphocytes, the mean counts of CD8^+^ T-lymphocytes were respectively 300.2, 506.3, and 484.3/μl in three groups, that in patients with twice or triple vaccination were elevated approximately 1.6-fold than patients without vaccination, and the percentage also showed a significant increase. The results indicated that vaccination could effectively increase CD8^+^ T-lymphocytes and eosinophils in COVID-19 patients.

### Blood biochemical profiles of COVID-19 patients

SARS-CoV2 infection may cause multiple organ involvement. Therefore we collected and analyzed biochemical indicators of all those patients (**Table 4**). There was no difference among the three groups studied for most liver functions, including GGT, ALT, AST, ALP, TP, A/G, TBA, LDH, and SOD. Bilirubin is a kind of bilins which is the main pigment in human bile and exists in two forms, namely direct bilirubin (D-Bil) and indirect bilirubin (I-Bil). The I-Bil levels of three-dose vaccinated patients were higher than in other groups, while the values of T-Bil and D-Bil showed no significant difference. In addition, three times vaccinated patients also had higher ADA levels. Similarly, almost all indicators of renal function tests exhibited no significant difference except serum sodium, including serum potassium, serum chlorine, CA, BUN, UA, and CR. Compared with vaccinated patients, the level of serum sodium in unvaccinated patients was significantly decreased.

**Table 4:** Blood biochemical profile analysis in patients with COVID-19.

	Normal range	Total (n = 231)	Unvaccinated (n = 21)	Two doses vaccinated (n = 158)	Three doses vaccinated (n = 52)	*P*-value	
γ-glutamyl transferase (GGT), U/L	10-60	17 (12-31)	26 (14.25-46)	18 (11.5-32.5)	13.5 (12-19.25)	*P*=0.2815	
Alanine aminotransferase (ALT), U/L	9-50	31 (23-42)	28 (22.25-48)	32 (25-42)	30.5 (23-39.5)	*P*=0.5137	
Aspartate aminotransferase (AST), U/L	15-45	29 (25-35)	27 (24-36)	29 (26-34)	27 (24.5-35.5)	*P*=0.3766	
Alkaline Phosphatase (ALP), U/L	45-125	68 (54.5-80)	71 (49-82)	67 (54-79.5)	68 (58.5-78.5)	*P*=0.7718	
Total bilirubin, μmol/L	0-26.0	9.8 (6.6-12.83)	8.2 (5.325-13.18)	9.7 (6.7-12.45)	11 (7.825-13.95)	*P*=0.1016	
Direct bilirubin (D-Bil), µmol/L	0-8.0	3.6 (2.8-4.7)	3.5 (2.425-4.4)	3.5 (2.8-4.7)	3.75 (2.8-4.85)	*P*=0.7093	
Indirect bilirubin (I-Bil), µmol/L	0-18.0	6 (3.95-8.4)	5 (2.5-7.6)	5.9 (3.9-7.9)	7.35 (5.1-10.03)	*P=0.0305	*P*1 = 0.7068, *P*2 = 0.0524, *P*3 = 0.1009
Serum albumin, g/L	40.0-55.0	45.8 (43.85-48)	45.7 (43.53-48.98)	45.65 (43.23-47.9)	46.85 (44.68-48.7)	*P*=0.1979	
Serum globulin g/L	20.0-40.0	24.25 (22-26.7)	24.9 (22.5-28.73)	24.35 (22.13-26.8)	23.75 (20.8-26.2)	*P*=0.1696	
Albumin/globulin (A/G)	1.2-2.4	1.9 (1.7-2.1)	1.9 (1.7-2.075)	1.8 (1.7-2.075)	2 (1.8-2.125)	*P=0.0337	*P*1>0.9999, *P*2 = 0.2676, *P3 = 0.0337
Total protein (TP), g/L	65.0-85.0	70.15 (66.3-73.8)	69.95 (64.73-75.6)	70.65 (66.4-73.6)	69.1 (66.8-74.43)	*P*=0.9654	
Adenosine deaminase (ADA), U/L	0-15	11 (9-13)	12.5 (10.25-20.5)	11 (9-13)	9.5 (8-11)	***P=0.0001	*P*1 = 0.2040, ***P2 = 0.0004, **P3 = 0.0014
Total biliary acid (TBA), µmol/L	0-12.0	2.86 (1.64-5.25)	2.91 (1.675-7.31)	2.825 (1.613-5.455)	3.62 (1.888-4.89)	*P*=0.9247	
Lactate dehydrogenase (LDH), U/L	120-250	186 (157-216)	190 (165-217)	187 (159-216)	165 (146.5-195.5)	*P*=0.2539	
Superoxide dismutase (SOD), U/L	129-216	151 (143.5-164)	145 (121-186)	149.5 (142.8-163.3)	156.5 (148-164)	*P*=0.3319	
Serum potassium (K), mmol/L	3.50-5.30	4.52 (4.18-4.89)	4.5 (4.018-5.03)	4.48 (4.12-4.93)	4.535 (4.338-4.863)	*P*=0.4822	
Serum sodium (Na), mmol/L	137.0-147.0	140.4 (139.1-142.2)	139.4 (137.9-140.8)	140.4 (139-142.3)	141.1 (139.7-142.4)	***P=0.001	**P1 = 0.0038, ***P2 = 0.0007, *P*3 = 0.5672
Serum chloride (CL), mmol/L	99.0-110.0	102.2 (100.5-103.9)	102.2 (99.15-102.9)	102.2 (100.5-103.9)	102.4 (101-104.2)	*P*=0.2325	
Serum calcium (Ca), mmol/L	2.11-2.52	102.2 (100.5-103.9)	102.2 (99.15-102.9)	102.2 (100.5-103.9)	102.4 (101-104.2)	*P*=0.2325	
Urea nitrogen (UN), mmol/L	3.1-8.0	3.7 (3-4.725)	4.95 (2.9-7.675)	3.5 (3-4.6)	3.9 (3.35-4.825)	*P=0.0280	*P*1 = 0.0605, *P*2 = 0.8934, *P*3 = 0.2673
Uric acid (UA), µmol/L	143-417	283 (223.8-347.5)	296.5 (217-358.8)	283 (216.3-328)	279.5 (237.8-380.8)	*P*=0.4517	
Creatinine (Cr), µmol/L	57-97	61 (50-72.25)	66 (52.25-77)	57 (49-69.75)	64.5 (56.75-77.25)	*P=0.0217	*P*1 = 0.3572, *P*2>0.9999, *P*3 = 0.4952

Continuous variables were shown in median (interquartile ranges). The Kruskal-Wallis test and post hoc Dunn’s test were used to analyze for the quantitative data of non-normal distribution among three groups. The One-way ANOVA and post hoc Bonferroni test were used to analyze for continuous variables normally distributed among three groups.

P: for three groups, P1: Unvaccinated vs. Two doses vaccinated, P2: Unvaccinated vs. Three doses vaccinated, P3: Two doses vaccinated vs. Three doses vaccinated.

*P < 0.05, **P < 0.01, ***P < 0.001.

## Discussion

Long-lasting high neutralizing antibody titers and immune memory are the sources of protection by COVID-19 vaccines that would provide sterilizing immunity to prevent disease and transmission (18). Studies have shown that both antibody responses and vaccine effectiveness after two doses of vaccine were higher than a single dose (19). However, IgA and IgG levels decreased significantly within 7 months after receiving the 2nd dose of SARS-CoV-2 vaccine, and after the booster vaccination, the antibody level increased again, to values higher than after the second dose (20). Our results showed that two and three doses of vaccination significantly increased the level of specific IgG in the serum of COVID-19 patients, which is consistent with the results of previous vaccine clinical trials (21, 22).

One of the most striking clinical features of COVID-19 patients is the dysregulated immune response, which leads to a cytokine storm, that is, the overproduction of proinflammatory cytokines (TNF-α, IL-1, and IL-6) and chemokines (IL-8) (23). The release of these factors causes acute activation of pulmonary vascular endothelial cells, infiltrating macrophages, and neutrophils, resulting in up-regulation of tissue factor expression in the alveoli, thereby amplifying the activation of the coagulation cascade effect (24). APTT, PT, and TT are the most commonly used intrinsic and extrinsic coagulation pathway screening tests. Previous research results showed that these indicators had no or only slight change in patients with COVID-19 (25, 26), which are more consistent with the hypercoagulable state in severe inflammation than disseminated intravascular coagulation (27). Our research showed that vaccination effectively shortened APTT and TT time, whereas the PT value did not change. Abnormal coagulation function and complications such as venous thromboembolism are the key influencing factors of poor prognosis and death in severe COVID-19 patients (28, 29). Vaccination can effectively prevent and alleviate the occurrence of abnormal coagulation and play a protective role in embolic complications.

Percopo H. F. et al. found that eosinophil had antiviral effects and promoted survival in mice infected with a lethal respiratory virus (30), and differences in eosinophils may be related to the protective effect of the vaccine. Consistent with previous results of eosinophilia in COVID-19 patients (31, 32), our findings showed that eosinophils were significantly higher in vaccinated patients than those in unvaccinated patients. Ren, X. et al. performed single-cell RNA sequencing on 284 samples from COVID-19 patients and controls and showed an elevated percentage of CD14^+^ monocytes in peripheral blood mononuclear cells, particularly in severe COVID-19 patients during the progressive disease stages (33). In the present study, the percentage of monocytes in vaccinated patients showed a significant downward trend, which may be related to the protective effect of the vaccine on cytokine storms frequently observed in severe patients.

Lymphocytes play a critical role in antiviral immunity, so it’s essential to clarify the changes in lymphocyte subsets in COVID-19 patients, which could provide new insights for exploring its immune mechanism. Many COVID-19 patients developed lymphopenia, especially in severe patients, indicating the damage to the immune system during the SARS-CoV-2 infection (34). Multiple studies have shown that lymphopenia is associated with a poor prognosis in COVID-19 patients (35–37). CD8^+^ T cells can directly attack and kill virus-infected cells through cytotoxicity (38). The study by Urra J. M. et al. showed that after SARS-CoV-2 infection, changes in CD8^+^ T cells were more pronounced than changes in other lymphocyte subtypes (39). Therefore, the decline of lymphocytes, especially CD8^+^ T cells, might be a potential predictor of COVID-19 disease severity and clinical deterioration. Our study found that vaccinated COVID-19 patients had significantly higher levels of CD8^+^ lymphocytes than unvaccinated patients, which is consistent with the vaccine’s protective effect, and this difference was not found between patients vaccinated with two and three doses.

Hyponatremia, defined as a plasma sodium concentration [Na^+^] <135 mEq/l, is one of the most common electrolyte disturbances in hospitalized patients (40). Pneumonia, cytokine release, and diuretic administration may all become the etiologies of hyponatremia in COVID-19 patients (41). Previous studies have demonstrated that nearly one-third of patients might develop hyponatremia, which was associated with an increased risk of encephalopathy and acute respiratory failure and was considered to be an independent predictor for COVID-19 progression to severe disease and death (41, 42). Our data suggested that vaccinated COVID-19 patients had higher plasma sodium concentrations than that of unvaccinated patients, which can prevent the occurrence of hyponatremia and related adverse prognosis. ADA is an enzyme involved in purine metabolism and plays a vital role in the maturation and maintenance of the immune system. ADA primarily catalyzes the deamination of adenosine, which acts as an immunosuppressive signal and prevents excessive inflammatory responses (43). At present, there are few studies on the changes of ADA in the serum of patients with COVID-19 and its impact on COVID-19 progression. In our results, vaccination significantly reduced the level of ADA in the patient’s serum, which may attenuate its regulatory effect on immunosuppression, but the specific mechanism needs to be further confirmed by more studies.

This study has several limitations that might lead to some potential biases. First, the patient sample from a single center is small, especially the sample of unvaccinated patients, and standardized data from larger cohorts will better assess the impact of vaccines on COVID-19. Second, the time interval between vaccination and SARS-CoV-2 infection varies in those patients with COVID-19. Since we did not have access to the time of the last dose of vaccine for all patients, the relationship between vaccination-infection interval and disease severity could not be assessed. Despite that, our study demonstrates that vaccines effectively protect against immune response and coagulation function dysregulation in COVID-19 patients.

## Data availability statement

The original contributions presented in the study are included in the article/**Supplementary Material**. Further inquiries can be directed to the corresponding authors.

## Ethics statement

The studies involving human participants were reviewed and approved by Institutional Ethics Committee of Xi’an Jiaotong University Health Science Center (No. 2022-1388). The patients/participants provided their written informed consent to participate in this study.

## Author contributions

Concept and design: XWL, YX, WZ, and LM. Data Acquisition: YX, DY, and WC. Interpretation of data and Manuscript drafting, including revisions: XWL, YX, XML, WL, DY, WC, HY, LH, and CJ. Statistical analysis: XML and WL. Supervision: SL, WZ, and LM. All authors contributed to the article and approved the submitted version.

## Funding

We are very grateful for the financial support from the National Natural Science Foundation of China (grant No. 82150201, 81970029, 82171784, and 82171724), China Postdoctoral Science Foundation (2021M702591), Fundamental Research Funds for the Central Universities (xpt012022036), and Xi’an Health Commission (COVID-19 special project).

## Conflict of interest

The authors declare that the research was conducted in the absence of any commercial or financial relationships that could be construed as a potential conflict of interest.

## Publisher’s note

All claims expressed in this article are solely those of the authors and do not necessarily represent those of their affiliated organizations, or those of the publisher, the editors and the reviewers. Any product that may be evaluated in this article, or claim that may be made by its manufacturer, is not guaranteed or endorsed by the publisher.

## References

[1] Holmes EC Goldstein SA Rasmussen AL Robertson DL Crits-Christoph A Wertheim JO. The origins of SARS-CoV-2: A critical review. Cell (2021) 184:4848–56. doi: 10.1016/j.cell.2021.08.017 PMC8373617 34480864

[2] Grenfell BT Pybus OG Gog JR Wood JL Daly JM Mumford JA. Unifying the epidemiological and evolutionary dynamics of pathogens. Science (2004) 303:327–32. doi: 10.1126/science.1090727 14726583

[3] Bakhshandeh B Jahanafrooz Z Abbasi A Goli MB Sadeghi M Mottaqi MS. Mutations in SARS-CoV-2; consequences in structure, function, and pathogenicity of the virus. Microb Pathog (2021) 154:104831. doi: 10.1016/j.micpath.2021.104831 33727169 PMC7955574

[4] Tian D Sun Y Zhou J Ye Q. The global epidemic of the SARS-CoV-2 delta variant, key spike mutations and immune escape. Front Immunol (2021) 12:751778. doi: 10.3389/fimmu.2021.751778 34917076 PMC8669155

[5] Harvey WT Carabelli AM Jackson B Gupta RK Thomson EC Harrison EM. SARS-CoV-2 variants, spike mutations and immune escape. Nat Rev Microbiol (2021) 19:409–24. doi: 10.1038/s41579-021-00573-0 PMC8167834 34075212

[6] Liu Y Rocklov J. The reproductive number of the delta variant of SARS-CoV-2 is far higher compared to the ancestral SARS-CoV-2 virus. J Travel Med (2021) 28:taab124. doi: 10.1093/jtm/taab124 34369565 PMC8436367

[7] Luo CH Morris CP Sachithanandham J Amadi A Gaston DC Li M. Infection with the SARS-CoV-2 delta variant is associated with higher recovery of infectious virus compared to the alpha variant in both unvaccinated and vaccinated individuals. Clin Infect Dis (2021) 75:e715–25. doi: 10.1101/2021.08.15.21262077 PMC8903351 34922338

[8] Pollard AJ Bijker EM. A guide to vaccinology: from basic principles to new developments. Nat Rev Immunol (2021) 21:83–100. doi: 10.1038/s41577-020-00479-7 33353987 PMC7754704

[9] Fiolet T Kherabi Y MacDonald CJ Ghosn J Peiffer-Smadja N. Comparing COVID-19 vaccines for their characteristics, efficacy and effectiveness against SARS-CoV-2 and variants of concern: a narrative review. Clin Microbiol Infect (2022) 28:202–21. doi: 10.1016/j.cmi.2021.10.005 PMC8548286 34715347

[10] Fang E Liu X Li M Zhang Z Song L Zhu B. Advances in COVID-19 mRNA vaccine development. Signal Transduct Target Ther (2022) 7:94. doi: 10.1038/s41392-022-00950-y 35322018 PMC8940982

[11] Hotez PJ Bottazzi ME. Whole inactivated virus and protein-based COVID-19 vaccines. Annu Rev Med (2022) 73:55–64. doi: 10.1146/annurev-med-042420-113212 34637324

[12] Tregoning JS Flight KE Higham SL Wang Z Pierce BF. Progress of the COVID-19 vaccine effort: viruses, vaccines and variants versus efficacy, effectiveness and escape. Nat Rev Immunol (2021) 21:626–36. doi: 10.1038/s41577-021-00592-1 PMC8351583 34373623

[13] Yewdell JW. Individuals cannot rely on COVID-19 herd immunity: Durable immunity to viral disease is limited to viruses with obligate viremic spread. PloS Pathog (2021) 17:e1009509. doi: 10.1371/journal.ppat.1009509 33901246 PMC8075217

[14] Hodgson SH Mansatta K Mallett G Harris V Emary KRW Pollard AJ. What defines an efficacious COVID-19 vaccine? a review of the challenges assessing the clinical efficacy of vaccines against SARS-CoV-2. Lancet Infect Dis (2021) 21:e26–35. doi: 10.1016/S1473-3099(20)30773-8 PMC7837315 33125914

[15] Rosenberg ES Holtgrave DR Dorabawila V Conroy M Greene D Lutterloh E. New COVID-19 cases and hospitalizations among adults, by vaccination status - new York, may 3-July 25, 2021. MMWR Morb Mortal Wkly Rep (2021) 70:1150–5. doi: 10.15585/mmwr.mm7034e1 PMC8389393 34437517

[16] Scobie HM Johnson AG Suthar AB Severson R Alden NB Balter S. Monitoring incidence of COVID-19 cases, hospitalizations, and deaths, by vaccination status - 13 U.S. jurisdictions, April 4-July 17, 2021. MMWR Morb Mortal Wkly Rep (2021) 70:1284–90. doi: 10.15585/mmwr.mm7037e1 PMC8445374 34529637

[17] Wei PF. Diagnosis and treatment protocol for novel coronavirus pneumonia (Trial version 7). Chin Med J (Engl) (2020) 133:1087–95. doi: 10.1097/CM9.0000000000000819 PMC7213636 32358325

[18] Sette A Crotty S. Adaptive immunity to SARS-CoV-2 and COVID-19. Cell (2021) 184:861–80. doi: 10.1016/j.cell.2021.01.007 PMC7803150 33497610

[19] Vasileiou E Simpson CR Shi T Kerr S Agrawal U Akbari A. Interim findings from first-dose mass COVID-19 vaccination roll-out and COVID-19 hospital admissions in Scotland: a national prospective cohort study. Lancet (2021) 397:1646–57. doi: 10.1016/S0140-6736(21)00677-2 PMC8064669 33901420

[20] Rastawicki W Juszczyk G Gierczynski R Zasada AA. Comparison of anti-SARS-CoV-2 IgG and IgA antibody responses post complete vaccination, 7 months later and after 3rd dose of the BNT162b2 vaccine in healthy adults. J Clin Virol (2022) 152:105193. doi: 10.1016/j.jcv.2022.105193 35660747 PMC9137250

[21] Al Kaabi N Zhang Y Xia S Yang Y Al Qahtani MM Abdulrazzaq N. Effect of 2 inactivated SARS-CoV-2 vaccines on symptomatic COVID-19 infection in adults: A randomized clinical trial. JAMA (2021) 326:35–45. doi: 10.1001/jama.2021.8565 34037666 PMC8156175

[22] Chagla Z. The BNT162b2 (BioNTech/Pfizer) vaccine had 95% efficacy against COVID-19 >/=7 days after the 2nd dose. Ann Intern Med (2021) 174:JC15. doi: 10.7326/ACPJ202102160-015 33524290

[23] Qin C Zhou L Hu Z Zhang S Yang S Tao Y. Dysregulation of immune response in patients with coronavirus 2019 (COVID-19) in wuhan, China. Clin Infect Dis (2020) 71:762–8. doi: 10.1093/cid/ciaa248 PMC7108125 32161940

[24] McGonagle D O'Donnell JS Sharif K Emery P Bridgewood C. Immune mechanisms of pulmonary intravascular coagulopathy in COVID-19 pneumonia. Lancet Rheumatol (2020) 2:e437–45. doi: 10.1016/S2665-9913(20)30121-1 PMC7252093 32835247

[25] Hadid T Kafri Z Al-Katib A. Coagulation and anticoagulation in COVID-19. Blood Rev (2021) 47:100761. doi: 10.1016/j.blre.2020.100761 33067035 PMC7543932

[26] Iba T Levy JH Levi M Connors JM Thachil J. Coagulopathy of coronavirus disease 2019. Crit Care Med (2020) 48:1358–64. doi: 10.1097/CCM.0000000000004458 PMC7255402 32467443

[27] Bonaventura A Vecchie A Dagna L Martinod K Dixon DL Van Tassell BW. Endothelial dysfunction and immunothrombosis as key pathogenic mechanisms in COVID-19. Nat Rev Immunol (2021) 21:319–29. doi: 10.1038/s41577-021-00536-9 PMC8023349 33824483

[28] Llitjos JF Leclerc M Chochois C Monsallier JM Ramakers M Auvray M. High incidence of venous thromboembolic events in anticoagulated severe COVID-19 patients. J Thromb Haemost (2020) 18:1743–6. doi: 10.1111/jth.14869 PMC7264774 32320517

[29] Cui S Chen S Li X Liu S Wang F. Prevalence of venous thromboembolism in patients with severe novel coronavirus pneumonia. J Thromb Haemost (2020) 18:1421–4. doi: 10.1111/jth.14830 PMC7262324 32271988

[30] Percopo CM Dyer KD Ochkur SI Luo JL Fischer ER Lee JJ. Activated mouse eosinophils protect against lethal respiratory virus infection. Blood (2014) 123:743–52. doi: 10.1182/blood-2013-05-502443 PMC3907759 24297871

[31] Xie G Ding F Han L Yin D Lu H Zhang M. The role of peripheral blood eosinophil counts in COVID-19 patients. Allergy (2021) 76:471–82. doi: 10.1111/all.14465 PMC7323233 32562554

[32] Rosenberg HF Foster PS. Eosinophils and COVID-19: diagnosis, prognosis, and vaccination strategies. Semin Immunopathol (2021) 43:383–92. doi: 10.1007/s00281-021-00850-3 PMC7962927 33728484

[33] Ren X Wen W Fan X Hou W Su B Cai P. COVID-19 immune features revealed by a large-scale single-cell transcriptome atlas. Cell (2021) 184:1895–1913.e19. doi: 10.1016/j.cell.2021.01.053 33657410 PMC7857060

[34] Wang D Hu B Hu C Zhu F Liu X Zhang J. Clinical characteristics of 138 hospitalized patients with 2019 novel coronavirus-infected pneumonia in wuhan, China. JAMA (2020) 323:1061–9. doi: 10.1001/jama.2020.1585 PMC7042881 32031570

[35] Tan L Wang Q Zhang D Ding J Huang Q Tang YQ. Lymphopenia predicts disease severity of COVID-19: a descriptive and predictive study. Signal Transduct Target Ther (2020) 5:33. doi: 10.1038/s41392-020-0148-4 32296069 PMC7100419

[36] Ruan Q Yang K Wang W Jiang L Song J. Correction to: Clinical predictors of mortality due to COVID-19 based on an analysis of data of 150 patients from wuhan, China. Intensive Care Med (2020) 46:1294–7. doi: 10.1007/s00134-020-06028-z PMC7080116 32125452

[37] Yang X Yu Y Xu J Shu H Xia J Liu H. Clinical course and outcomes of critically ill patients with SARS-CoV-2 pneumonia in wuhan, China: a single-centered, retrospective, observational study. Lancet Respir Med (2020) 8:475–81. doi: 10.1016/S2213-2600(20)30079-5 PMC7102538 32105632

[38] Rydyznski Moderbacher C Ramirez SI Dan JM Grifoni A Hastie KM Weiskopf D. Antigen-specific adaptive immunity to SARS-CoV-2 in acute COVID-19 and associations with age and disease severity. Cell (2020) 183:996–1012.e19. doi: 10.1016/j.cell.2020.09.038 33010815 PMC7494270

[39] Urra JM Cabrera CM Porras L Rodenas I. Selective CD8 cell reduction by SARS-CoV-2 is associated with a worse prognosis and systemic inflammation in COVID-19 patients. Clin Immunol (2020) 217:108486. doi: 10.1016/j.clim.2020.108486 32479985 PMC7256549

[40] Henry DA. In the clinic: Hyponatremia. Ann Intern Med (2015) 163:ITC1–19. doi: 10.7326/AITC201508040 26237763

[41] Berni A Malandrino D Corona G Maggi M Parenti G Fibbi B. Serum sodium alterations in SARS CoV-2 (COVID-19) infection: impact on patient outcome. Eur J Endocrinol (2021) 185:137–44. doi: 10.1530/EJE-20-1447 PMC9494309 33950864

[42] Frontera JA Valdes E Huang J Lewis A Lord AS Zhou T. Prevalence and impact of hyponatremia in patients with coronavirus disease 2019 in new York city. Crit Care Med (2020) 48:e1211–7. doi: 10.1097/CCM.0000000000004605 PMC7467047 32826430

[43] Gao ZW Wang X Zhang HZ Lin F Liu C Dong K. The roles of adenosine deaminase in autoimmune diseases. Autoimmun Rev (2021) 20:102709. doi: 10.1016/j.autrev.2020.102709 33197575

