# Supplementary material for: Real-world effectiveness and protection of SARS-CoV-2 vaccine among patients hospitalized for COVID-19 in Xi’an, China, December 8, 2021, to January 20, 2022: A retrospective study
Source: Front Immunol. 2022 Sep 23;13:978977. doi: 10.3389/fimmu.2022.978977 (PMC9538118; doi:10.3389/fimmu.2022.978977)

Fig. S1. Sample collection time and symptom onset time analysis.

A. Time from symptom onset to hospital admission. B. Time from admission to SARS-CoV-2 antibody detection. C. Time from admission to lymphocyte subtype detection. Statistical significance was determined by One-way ANOVA and post hoc Bonferroni test. ns: no significant.

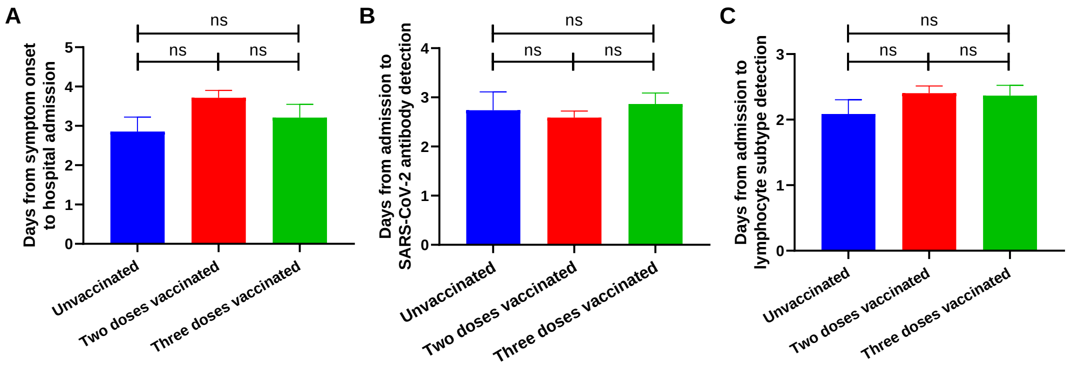

Supplement: Supplementary file 1 [file DataSheet_1.pdf]
